# Effects of rehabilitation approaches for runners with patellofemoral pain: protocol of a randomised clinical trial addressing specific underlying mechanisms

**DOI:** 10.1186/s12891-015-0859-9

**Published:** 2016-01-06

**Authors:** Jean-Francois Esculier, Laurent J. Bouyer, Blaise Dubois, Pierre Frémont, Lynne Moore, Jean-Sébastien Roy

**Affiliations:** Faculty of Medicine, Laval University, 1050 Avenue de la Médecine, Quebec City, QC G1V 0A6 Canada; Centre for Interdisciplinary Research in Rehabilitation and Social Integration, 525 Boulevard Wilfrid-Hamel, Quebec City, QC G1M 2S8 Canada; The Running Clinic, C.P. 1075, Lac-Beauport, QC G3B 2J8 Canada

**Keywords:** Patellofemoral, Knee pain, Running, Exercises, Gait retraining, Physical therapy

## Abstract

**Background:**

Patellofemoral pain (PFP) is highly prevalent in runners, and often leads to functional limitations and cessation of running. Training errors as well as decreased lower limb strength and control during running have all been associated with PFP. While strengthening and gait retraining are commonly used by clinicians, no randomised clinical trial has compared these modalities in runners with PFP. The primary objective of this randomised clinical trial will be to compare the effects of three rehabilitation programs targeting different key factors on symptoms and functional limitations of runners with PFP. The secondary objective will be to explore the factors leading to clinical improvement.

**Methods/design:**

We will conduct a single-blind randomised clinical trial to compare three different 8 week rehabilitation programs: Group 1 will receive education on symptoms management based on training modifications; Group 2 will receive an exercise program targeting lower limb strengthening and control in addition to the education component of Group 1; Group 3 will receive running gait retraining advice as well as the education component of Group 1. Sixty-nine runners with PFP will be recruited and will be seen by independent physiotherapists on five visits through 8 weeks. The primary outcome measure will be symptoms and functional limitations measured by the Knee Outcome Survey – Activities of Daily Living Scale questionnaire at baseline, and at the four, eight and 20 weeks follow-up. Secondary outcomes will include pain level measured using visual analog scales, and running mileage. Lower limb kinematics and kinetics during running, and isometric strength will also be evaluated at baseline and 8 weeks follow-up. The effects of rehabilitation programs on measures of symptoms and functional limitations will be assessed using a 2-way ANOVA (Groups x Time). Regression analyses will be used to identify if changes in running mechanics or strength are determinants of clinical success.

**Discussion:**

Studies with a high level of evidence are needed to determine the best rehabilitation interventions for runners with PFP. This randomised clinical trial will be the first to compare programs targeting different key factors linked with PFP. Results may guide clinicians and improve their clinical outcomes when treating runners with PFP.

**Trial Registration:**

ClinicalTrials.gov: NCT02352909. Registered on December 3, 2014.

## Background

Recreational running has gained popularity among the general population due to its ease of access. Despite all the benefits of recreational running, musculoskeletal injuries are frequent in runners with a reported prevalence of up to 79 %, the knee being the most affected joint. [1] Patellofemoral pain (PFP) has been reported as the most frequent running injury, ranging from 19 to 30 % of diagnostics in women, and 13 to 24 % in men [2, 3]. Thus, it is not surprising that PFP is also known as “Runner’s knee” [4].

Running-related PFP is thought to be caused by a combination of factors that can be related to training errors or to inadequate lower limb muscle strength or control [5–7]. Since running is associated with tremendous cumulative loads in lower limb joints, training errors are believed to be partly responsible for over 60 % of running-related injuries [5, 7, 8]. Clinical observations reveal that runners diagnosed with PFP often had recently incorporated changes in their training regimen such as increases in running mileage or speed, as well as rapid additions of downhill and stairs running [9, 10]. Education on appropriate training loads to respect the envelope of function should therefore be addressed as a main component of interventions in runners with PFP [11, 12].

Several studies have reported that PFP in runners could be related to deficits in lower limb strength. Cross-sectional studies suggest that runners with PFP may present with strength deficits in their knee extensors [13] or hip abductors [14, 15] compared with runners without PFP. However, in two prospective studies involving participants in a start-to-run program, the 21 runners who developed PFP did not have a lower limb strength deficit to these muscle groups prior to their enrolment in the training program [16, 17]. Thus, it can be hypothesised that strength deficits in individuals with PFP may be a result of pain rather than a cause [18].

Lower limb control deficits during running have also been identified in runners with PFP. Noehren et al. found that female runners with PFP exhibited increased peak angles of hip adduction and internal rotation during the stance phase of running compared with healthy female runners [19]. In addition, Willy et al. observed that male runners with PFP had significantly more contralateral pelvic drop than healthy controls, but also showed significantly less hip adduction compared with females with PFP [20]. Based on analyses using dynamic magnetic resonance imaging [21], it could be hypothesised that increased peak hip adduction, hip internal rotation and contralateral pelvic drop during running would result in focal retropatellar overload. Excessive patellofemoral joint forces during running could also potentially be the result of higher ground reaction forces. Specifically, the vertical loading rate of the impact force has been associated with several injuries secondary to a running program [22–24], including PFP [25].

Based on the data presented above, several factors should be considered when selecting a rehabilitation program in runners with PFP. First, the program could simply focus on modifying training loads in order to manage pain and promote tissue adaptation within the envelope of function [11]. Second, exercises targeting the quadriceps as well as hip and core muscles [26] could be prescribed to increase the capacity to sustain loads by strengthening or improving lower limb control. The program could also include gait retraining to decrease forces at the knee during running. Proposed interventions to achieve this goal include increasing step frequency [27], running softer [28] and avoiding a rearfoot strike pattern [29].

To our knowledge, there have been six published studies on rehabilitation interventions in runners with PFP. A recent study from our research group [30] found that an 8 week multimodal program combining education, an exercise program and gait retraining was successful in reducing symptoms and improving function (*n* = 21). Interestingly, reduction in the vertical loading rate was found to be a key factor for improvement. Four other laboratory studies have used gait retraining for runners with PFP. First, Cheung & Davis (*n* = 3) reached clinical success after using feedback for vertical loading rate while runners were asked to avoid a rearfoot strike pattern [31]. This decrease in impact forces was linked with improvements in pain levels and self-reported function. Noehren et al. (*n* = 10) and Willy et al. (*n* = 10; *n* = 2) used a similar training protocol, but provided feedback on peak hip adduction using either a motion analysis system or a mirror [32–34]. In addition to reductions in peak hip adduction angle following gait retraining, participants improved their scores on pain scales. Interestingly, significant reductions in vertical loading rate were also found in Noehren et al’s study [32]. Finally, another study (*n* = 15) reported improvement in symptoms and functional status as well as hip abductor strength following 3 weeks of hip strengthening exercises [15].

While different rehabilitation approaches addressing deficits in runners with PFP show some success in studies with level of evidence of two or three, it is not clear which one is more effective, or why. In fact, no randomised clinical trial has been performed in this population. The primary objective of this randomised clinical trial will be to compare the effects of three rehabilitation programs on symptoms and functional limitations of runners with PFP. The programs will include either education alone, a combination of education and exercises, or a combination of education and gait retraining. The secondary objective will be to identify the factors leading to clinical improvement. Our general hypothesis is that all three interventions will be efficient in decreasing symptoms and improving function. However, based on previously published research in this population, we believe that the group including gait retraining will experience greater and faster improvements than the two other groups. We also hypothesise that changes in running gait pattern associated with a decrease in knee loading and vertical loading rate will be responsible for the greater improvement.

## Methods/design

### Study design

This stratified, single-blind, parallel-group randomised clinical trial will include four evaluation sessions (baseline, week 4, week 8, week 20) and five supervised physiotherapy sessions during an 8 week rehabilitation program. The evaluation sessions will be carried out at the Centre for Interdisciplinary Research in Rehabilitation and Social Integration (CIRRIS) in Quebec City, Canada. The supervised physiotherapy treatments will be conducted at the *Clinique de physiothérapie PCN La Capitale*, a private clinic specialised in the treatment of running injuries. Runners will be allocated to either 1- an intervention that will only include education and training modifications according to symptoms; 2- the addition of a strengthening and lower limb control exercise program to the education component; 3- modifications to the running gait in addition to the education component.

### Participants

Sixty-nine recreational runners with PFP will be recruited through advertisements in running events and sports medicine clinics in Quebec City, Canada, as well as through emails sent to Laval University students and employees. To be included, potential participants will have to meet the following inclusion criteria: 1- age between 18 and 45 years; 2- run at least 15 km per week; 3- present a history of anterior knee pain for at least 3 months; 4- report a pain level of at least 3/10 on a visual analog scale during running and during at least three activities among: kneeling, squatting, stairs and resisted knee extension [35]; and 5- score lower than 85/100 on the Knee Outcome Survey – Activities of Daily Living Scale questionnaire (KOS-ADLS), to ensure a minimum level of symptoms and limitations (clinically important difference of 13.6 points) [36]. In addition, runners will be excluded if they present one of the following conditions: 1- history of lower limb surgery or patellar dislocation; 2- pain believed to originate either from meniscus [37, 38] or from patellar tendon [39]; 3- pain following an acute trauma; 4- concurrent lower limb injuries; 5- history of neurological, inflammatory or rheumatoid disease. A member of the research team will perform a preliminary screening of the inclusion and exclusion criteria and explain the study during a telephone call. If they agree to participate, potential participants will then be met by a physiotherapist to confirm the diagnosis of PFP using the clinical tests. Ethics approval was obtained from the Institutional Review Board of Quebec Rehabilitation Institute (#2014-367), and all subjects will sign a detailed consent form before entering the study.

### Procedures

All included runners will take part in the baseline evaluation session. First, questionnaires on sociodemographic, symptomatology, comorbidity, and running habits will be completed. Thereafter, the level of symptoms and functional limitations will be evaluated using self-reported questionnaires (KOS-ADLS and visual analog scales), before assessing knee and hip muscle strength. Then, lower limb kinematics and kinetics will be evaluated while running on an instrumented treadmill. Finally, runners will be randomly assigned to one of the three intervention groups, and will take part in their assigned 8 week rehabilitation program.

At weeks 4, 8, and 20 (3 months following completion of the program), self-reported questionnaires completed at baseline will be re-administered. This will allow us to determine if a program leads to a faster recovery and to better long-term effects. At week-8 evaluation, lower limb mechanics during running and strength will also be revaluated in the laboratory. All runners will be provided with a GPS-enabled watch (Garmin Forerunner 15) so that running mileage, speed, duration and step count are monitored during the intervention period.

### Randomisation/blinding

A randomisation list will be generated by an independent research assistant (not involved in data collection) using a computer random number generator. Allocation will be concealed in sequentially numbered sealed opaque envelopes. Given the influence of gender and foot strike pattern on lower limb mechanics during running, randomisation will be stratified to ensure balance of the treatment groups with respect to these variables (male/female; rearfoot strikers/non-rearfoot strikers). A block randomisation will also be used to make sure that three equal groups of 23 runners are obtained. Given that it is impossible to blind the treating physiotherapists and the runners, a single-blind design will be used, in which the evaluator will be blinded. Participants will be instructed not to discuss the specific program received with the evaluator.

### Outcome measures (dependent variables)

We chose the KOS-ADLS as the primary outcome to monitor clinical improvement. Secondary outcomes include VASs, a Global Rating of Change (GRC) question, as well as lower limb isometric muscle strength and running kinematics and kinetics.

#### Knee outcome survey – activities of daily living scale

A systematic review pertaining to self-reported questionnaires used in individuals with PFP recommended the KOS-ADLS over other knee-specific scales based on its psychometric properties and clinical applicability for runners [40]. The KOS-ADLS is a 14-item knee-specific questionnaire that evaluates symptoms and functional limitations experienced during activities of daily living in individuals with various knee disorders [41]. The validated French version of the KOS-ADLS will be used (MDC90 = 8.3 points; CID = 13.6 points) [36].

#### Visual analog scales

Knee pain will be assessed with VASs for usual pain, worst pain and pain during running. These VASs were shown to be reliable and responsive in runners with PFP [36].

#### Running mileage

Runners’ training will be monitored using a GPS-enabled Garmin Forerunner 15 watch. Weekly running mileage will be made available to researchers using an online log, and will allow us to assess changes in training volume during the 8 week intervention.

#### Lower limb isometric strength

Strength will be assessed by producing maximal voluntary isometric contraction of knee extensors, hip external rotators, abductors and extensors using a Medup handheld dynamometer (Atlas-Medic, Quebec City, Canada) as per validated methods [42]. Non-elastic straps will be used to secure participants’ position and limbs during testing. The peak force value (kg) from three trials will be considered and normalised in percentage of bodyweight [43, 44].

#### Lower limb kinematics and kinetics

Lower limb mechanics of the affected leg during running will be evaluated using kinematic and kinetic data. Rigid non-colinear triads of reflective markers will be placed over the cervicothoracic and lumbosacral junctions as well as bilaterally on the lateral part of the foot, shank and thigh. Triads attached to the thighs will be made of custom-molded thermoplastic, and securely attached using Velcro straps to minimize movement artefacts. Anatomical markers will be temporarily installed for a standing calibration trial at the anterior and posterior tips of the shoes, lateral head of the fifth metatarsals, medial and lateral malleolli, medial and lateral femoral condyles, iliac crests, anterior superior iliac spines and lateral tip of the acromion. Kinematic data will be collected at 200 Hz using an 8-camera VICON MX-T motion capture system and VICON Nexus software (VICON motion systems, CA, USA). All participants will start by running on an instrumented treadmill (Bertec Corp, Columbus, OH) during 5 min to ensure consistency of running kinematics. To reproduce individual training conditions, runners will wear their usual running shoes and will select their preferred running speed between eight and 10 km/h (same shoes/speed for weeks 0 and 8) [45]. Running data will then be collected during the following 3 min.

Kinematic variables of interest will be hip adduction, hip internal rotation as well as contralateral pelvic drop excursions and peak angles during the stance phase [19, 20]. Kinetic variables of interest will include the vertical loading rate [46], and maximum vertical ground reaction force. In addition, Newton–Euler inverse dynamics equations will be used to estimate the shear and compression reaction forces within the sagittal plane at the patellofemoral, tibiofemoral, and ankle joints [47, 48].

### Rehabilitation programs (independent variables)

Each runner will take part in one of three rehabilitation programs carried out over five sessions by independent physiotherapists. A training session will be performed prior to recruitment to ensure standardisation of the intervention between the physiotherapists, and detailed documents reviewing the content of each meeting will be provided. Furthermore, frequent follow-ups will be made by the research team with the physiotherapists to ensure the consistency of program delivery. Runners will receive written instructions, and, when applicable, pictures illustrating the exercises. All components of each intervention will be addressed at every visit to the clinic. During the programs, participants will continue to run.

A) *Education group*
  This group will receive a program that will only include the same education component on symptoms management that the two other groups will receive. Runners will be asked to run more often, but to decrease daily distance and running speed as well as to avoid downhill running. They will be instructed to maintain patellofemoral pain level at a maximum of 2/10 during running. In addition, pain should return to before-training levels within 60 min post training. Gradually, running distance will be increased according to symptoms, up to baseline level or more, before adding speed and hills [30].
B) *Exercise group* (strengthening and lower limb control)
  In addition to the education on symptoms management, runners will be asked to perform a standardised exercise program focusing on strengthening and lower limb control three times per week. The same exercise program was used in a previous study from our research group [30]. Each exercise session will last a maximum of 20 min. Exercises will be gradually progressed through higher difficulty levels of knee/hip/core strength and lower limb control including jumps and elastic bands. Exercises will be individually dosed and progressed by the treating physiotherapist. No recommendation about lower limb alignment during running will be provided.
C) *Gait retraining group* (reducing impact forces)
  In addition to education on pain management, runners will receive personalised advice on running mechanics in order to reduce patellofemoral joint load. Specifically, step rate will be increased, and noise produced during running will be decreased so that vertical ground reaction forces are reduced [28, 29, 49–51]. Runners for whom noise reduction will not be an efficient strategy will be asked to modify their foot strike pattern to midfoot or forefoot [31]. We decided to use the approach targeting decreased ground reaction forces since it is applicable to a vast majority of runners, compared with the kinematics approach which has only been tested in runners with excessive hip adduction [32, 34].

Sample size calculation and statistical analyses: Sample size calculation is based on changes to KOS-ADLS scores, our primary outcome, which are expected to increase following the different interventions. In a previous study using an 8 week multimodal intervention program similar to the current study, KOS-ADLS scores increased from 71.7 ± 12.9 to 89.5 ± 11.9 [30]. Considering similar score changes to our primary outcome, α = 0.05, power of 90 % and CID of 13.6 points for the KOS-ADLS, 20 runners are needed in each group (G*Power 3.1.7). When adding an anticipated attrition rate of 15 %, 23 runners per group are needed; therefore, a total of 69 runners will be recruited. Baseline demographic data will be compared (one-way ANOVA and Chi-squared tests). If baseline characteristics are different across groups, we will adjust group comparisons for confounding variables using an ANCOVA model. An intention-to-treat analysis will be used in which all participants will be analysed in the group to which they were originally assigned, up to the 8 week point. Dropouts or loss to follow-up will not be carried forward to the 3 month follow-up, since a successful outcome after 8 weeks may not necessarily be maintained 3 months later. This will avoid false assumptions regarding durable benefits from the intervention. Per protocol analyses (i.e., analyses restricted to patients who received the intervention as intended) will also be performed. All dropouts and reasons underlying dropouts will be reported. If requested, subjects electing to drop-out will be referred to their treating physician for other treatment options. Any harms or unintended effects during the course of the rehabilitation programs will be reported and discussed. For objective one, a 2-way ANOVA (Groups x Time) will be used to analyse the effects of the rehabilitation programs on KOS-ADLS, VASs and running mileage. For objective two, logistic regression analyses will be performed to identify if changes in running mechanics or strength are determinants of clinical success. For these analyses, results to KOS-ADLS will be dichotomised to changes equal or superior to the CID of 13.6 points, or to changes inferior to this value [36]. Subjective success will also be dichotomised to GRC ≥ +5 or GRC < +5.

## Discussion

Regular physical activity provides considerable physical and psychological health benefits [52]. Unfortunately, injuries often lead to a significant decrease or even cessation of recreational running, which could potentially be detrimental for health. Growing interest in recreational running and the relatively high incidence of PFP in this population suggest that the number of runners suffering from PFP will continue to rise in the upcoming years. Intervention studies with a high level of evidence are needed to improve the efficiency of rehabilitation. Despite several studies looking at factors associated with PFP in runners, few intervention studies addressing these factors with adequate sample size have been performed. This randomised clinical trial will be the first study to evaluate the added value of an exercise program or gait retraining to education. The results from this study will help physiotherapists and sports medicine practitioners improve clinical outcomes in runners with PFP.

## Abbreviations

ANOVA: Analysis of variance
CID: Clinically important difference
CIRRIS: Centre for interdisciplinary research in rehabilitation and social integration
GRC: Global rating of change
KOS-ADLS: Knee outcome survey – activities of daily living scale
MDC: Minimal detectable change
PFP: Patellofemoral pain
VAS: Visual analog scale
